# Day to Day Clinically Relevant Corneal Elevation, Thickness, and Curvature Parameters Using the Orbscan II Scanning Slit Topographer and the Pentacam Scheimpflug Imaging Device

**DOI:** 10.4103/0974-9233.61216

**Published:** 2010

**Authors:** Hassan Hashemi, Shiva Mehravaran

**Affiliations:** 1 Noor Ophthalmology Research Center, Noor Eye Hospital, Tehran, Iran; 2 Farabi Eye Hospital, Tehran University of Medical Sciences, Tehran, Iran

**Keywords:** Corneal Curvature, Corneal Elevation, Orbscan, Pentacam, Posterior Ectasia, Scheimpflug, Slit Scanning, Topography

## Abstract

The introduction of different techniques and computerized devices into clinical ophthalmology has significantly improved our knowledge of the eyes, optics, and eye conditions. Today, corneal topography is performed with a wide range of devices that implement a variety of techniques. Advance computerized analysis systems provide us with simple and quick evaluation procedures, yet the sophisticated data and clinical information that is generated can only be interpreted with adequate knowledge of the system itself as well as the accepted normal ranges of various properties assessed with these systems. Two computerized topography systems that are in common use are the Orbscan (Bausch and Lomb Inc., Rochester, NY, USA) and the Pentacam (Oculus GmBH, Wetzlar, Germany). The Orbscan is a slit-scanning device and the Pentacam is Scheimpflug imaging device. In this review, we present a brief description of both technologies, the techniques implemented in each device and the acquisition process with each. This will be followed by a list of corneal parameters that need to be assessed in screening patients for refractive surgery. We will discuss how these parameters are displayed, how each parameter may serve as clinic criteria, and how data should be interpreted. We will also try to provide evidence regarding the accuracy of different measurements, and the comparability of the two devices.

## INTRODUCTION

In 1619 Scheiner provided the first accurate description of the anatomy of the eye, as well as the corneal shape using glass balls of known curvatures^1^. Since then, different corneal assessment tools have been devised which include, the keratometer, the ophthalmometer, the Placido disc, and the photokeratoscope. Today, these devices have evolved into sophisticated computerized systems, generally categorized into reflection-based and projection-based corneal topographers. Some are capable of reconstructing the three dimensional image of the anterior segment of the eye, and generate a tremendous amount of data within seconds with minimal patient discomfort.

In many clinical settings, we see these advanced topographers next to simple manual keratometers that measure the curvature of the cornea at two principal meridians.

Perhaps one of the most common applications of topographers, regardless of their level of sophistication, is screening patients for keratorefractive procedures. The screening process should allow the physician to identify disease conditions, such as corneal ectatic disorders, that are contraindications for corneal laser refractive procedures. Even in the absence of disease conditions, there are certain variables that, individually or collectively, determine whether the patient is eligible for laser *in situ* keratomileusis (LASIK), photorefractive keratectomy (PRK), or non-laser treatment. To choose the most appropriate treatment, data on a number of variables such as refraction, keratometry and pachymetry, are checked against a list of criteria.

Another important application of corneal topographers is the diagnosis of corneal conditions that affect the normal curvature and/or thickness of the cornea and follow up. Keratoconus, for example, is defined as non-inflammatory progressive corneal thinning that causes irregular astigmatism, myopia, protrusion of the cornea in the shape of a cone, and impaired quality of vision. When examined with a hand-held keratoscope, keratoconus appears as an inferior or inferotemporal deviation of the horizontal axis and compression of mires.^2^ Videokeratoscopy allows quantitative assessment of the corneal curvature several topographic criteria that can be used for the diagnosis of keratoconus.^3^ Data generated from advanced computerized systems, however, is not limited to keratometry readings, and various aspects of corneal shape can be examined such as curvature, elevation, and thickness. Two popular examples of such topography systems are the Orbscan (Bausch and Lomb Inc., Rochester, NY, USA) and the Pentacam (Oculus GmBH, Wetzlar, Germany) both of which have a variety of applications. In the current article we review the use of the Orbscan and Pentacam in the field of refractive surgery.

## ORBSCAN

The original version of Orbscan slit-scanning topography system was launched in 1995. At that time, Orbscan was the only commercially available machine that could measure surface elevation of the cornea. In the later version, namely the Orbscan II, the Placido disc technique was incorporated to enable direct measurement of corneal curvature. Some of the most important variables that are measured indirectly include the corneal thickness and anterior chamber depth.^4^

**Figure 1:**
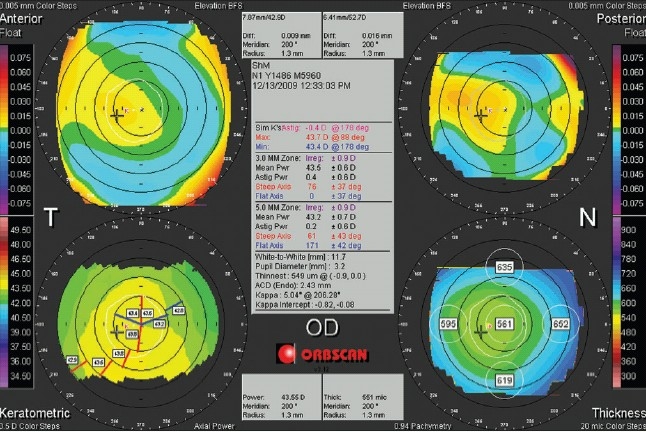
An Orbscan Quadmap of a normal right eye. The four maps, clockwise, from the top left include the anterior elevation, posterior elevation, pachymetry, and axial power. See text for more information

The examination process with the Orbscan, similar to other computerized topography systems, begins with entering basic information, proper positioning of patient head, forehead, and chin, and adjusting the instrument. During data acquisition, 20 slits are projected onto the cornea from each side for a total of 40 slits. This is done in a scanning fashion at an angle of 45 degrees, and the backscattered light is captured by a digital video camera. Data from 240 points are extracted from each slit, and processed by the software to calculate different variables.^5^,^6^ The most common display is the “quadmap” that includes 2-dimmensional color-coded maps of the anterior and posterior corneal surface elevation, the corneal thickness or pachymetry map, and the corneal curvature or power map [Figure 1]. In Figure 1, the four maps, clockwise, from the top left include the anterior elevation, posterior elevation, pachymetry, and axial power. The top middle gray boxes displays the best fit sphere diameter/power of their adjacent map, the elevation reading at the location of the cursor, and the meridian and radius at the location of the cursor. The information in the middle gray box, from top, includes patient name and ID, the exam date, and simulated keratometry readings. The irregularity index value and power data of the 3.0 and 5.0 mm zones follow below. The bottom section displays the corneal diameter, pupil diameter, location and pachymetry of the thinnest point on the cornea, the anterior chamber depth (ACD), and the angle kappa size and intercept. The left and right bottom two boxes present the corneal power and corneal thickness at the location of the cursor, respectively.

The Orbscan gained instant popularity, and numerous research projects were conducted to assess its measurement accuracy and clinical applications. In the following section we will review some of the variables and maps that are commonly assessed for screening refractive surgery candidates.

### Best fit sphere

Similar to terrain topography, where surface elevation is surveyed in reference to sea level, corneal surface elevation is measured from a reference, however, the reference is not fixed. Although the cornea is not exactly spherical in shape, the most commonly used reference surface is a sphere that freely adjusts its diameter and position to fit the given corneal surface with minimum square difference. This is called the floating best fit sphere (BFS).

In corneal ectasia, the earliest signs presumably occur in the posterior cornea. A posterior BFS value more than 51.0 D has been suggested as an indicator of primary posterior corneal elevation, and a value more than 55.0 D is a criterion for the diagnosis of forme fruste keratoconus (FFKC). The ratio of radii of the anterior BFS to the posterior BFS should also be considered. A ratio below 1.21 is acceptable, whereas a ratio greater than 1.27 is a contraindication for excimer laser refractive surgery. Eyes that have ratios between 1.21 and 1.27 should be regarded as keratoconus suspects and treated with caution.^7^,^8^

### Surface elevation maps

Orbscan generates elevation data of the anterior and posterior corneal surfaces, as well as the anterior iris, and the anterior lens as far as the pupil allows. The common display for this information is the two dimensional color-coded map, where green represents points very close to the reference surface or the BFS, warmer colors represent points above the BFS, and cooler colors are areas below the BFS.

**Figure 2:**
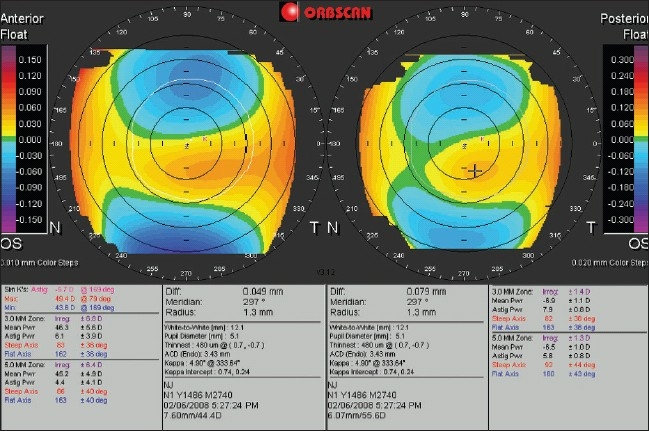
An Orbscan dual display of a keratoconic left eye. The anterior elevation map (left) is displayed on a 10 µm (0.010 mm) step scale and the posterior elevation map is on a 20 µm step scale. In both maps, there are more than 3 colors in the central zone; a sign indicative of keratoconus. Note that larger scale steps would increase the scale range and show abnormal areas with less alarming colors (See Figure 3).

To understand the shape of a given cornea and rule out abnormalities, the anterior elevation map and more specifically, the posterior elevation map should first be studied in their entirety. Elevation patterns are classified as regular ridge, irregular ridge, incomplete ridge, island, and unclassified. Although reported frequencies vary greatly, common patterns seen on anterior elevation maps are. island and incomplete ridge, and the most common pattern on posterior elevation maps is the island.^6^,^9^ In this process, we can identify abnormally elevated areas, or asymmetric patterns of corneal elevation, such as inferior or inferotemporal deviation of the area of maximum elevation. The accuracy of such observations depends on the choice of the color scale and the step size. Tanabe *et al*.^10^ suggest using 10 and 20 µm interval color scales on the anterior and posterior elevation maps, respectively. Using Tanabe's recommendation, maps with more than three colors in the central 3.0mm are considered abnormal [Figure 2].

**Figure 3:**
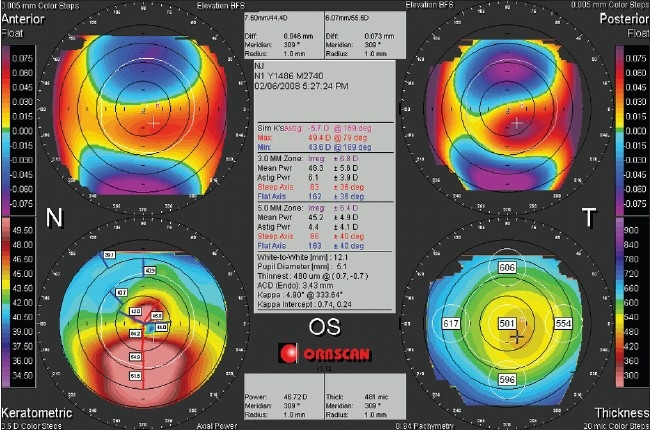
The Orbscan quadmap of the keratoconic eye in figure 2. Both elevation (anterior, top right; posterior, top left) maps are set on a 5µm step scale, and the abnormally elevated parts in the center are mapped with red. Note the best fit sphere diameter/power (7.60 mm /44.4 D anterior, 6.07 mm /55.6 D posterior) and the high elevation readings at the point of the cursor (46 µm anterior, 73 µm posterior)

Quantitative criteria should be checked as well. According to Rao *et al.*,^11^ keratoconus suspect eyes have a mean anterior elevation of 10 µm, and a mean posterior elevation of 35 µm, compared to 5µm and 21µm, respectively, in normal controls. In keratoconic eyes, posterior elevation readings in the cone area are greater than 40 µm [Figure 3]. In distinguishing keratoconus and keratoconus suspects from normal, Fam *et al.*,^12^ report that a posterior elevation of 40 µm or more has a sensitivity of only 57.7% and a specificity of 89.9%, and suggest using anterior corneal parameters, such an anterior elevation ratio (anterior elevation/anterior BFS) of 0.5122 or less, instead of posterior elevation.

**Figure 4:**
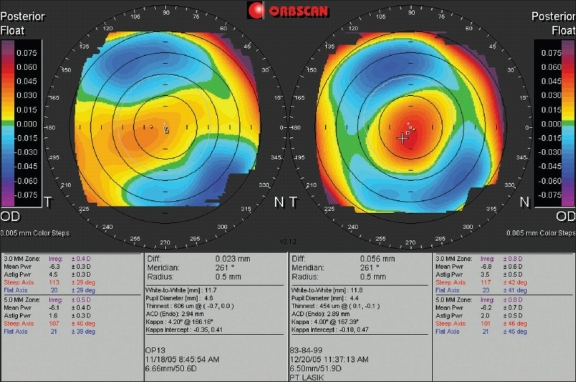
A dual posterior elevation display of a right eye before (left) and 6 weeks after (right) keratorefractive surgery for myopia. The scale step and range in these two maps are similar. Note the change in central zone posterior elevation which can be interpreted as posterior corneal bulging or a forward shift

The continued use of the Orbscan allowed studies of corneal changes after refractive surgery. The Orbscan comes with a feature that allows comparison of two maps, and a difference display that subtracts readings on corresponding points and plots them on one map. As one would expect, the greatest amount of change of anterior elevation after corneal laser treatment for myopia appear centrally and for hyperopia appear in the peripheral treatment zones. Research with the Orbscan on the posterior corneal surface demonstrated significant levels of posterior bulging or forward shift after refractive surgery [Figure 4]. Initially, this was considered a potential sign of ectasia.^13^–^16^ however further research provided evidence that the observation was an artifact, and likely due to changes in the corneal magnification effect.^17^–^20^

### Corneal thickness

Another feature of the Orbscan, which was initially quite awe-inspiring for users, was a pachymetry map of the entire corneal surface. The data are normally displayed as a color-coded map where green represents normal ranges of corneal thickness, purple and warm colors indicate thicker areas of the cornea, and red is used to display alarmingly thinner areas. To facilitate a quick review, the common display includes numeric values in 5 points that include the center of the cornea, and the superior, inferior, nasal, and temporal points 3.0 mm from the center, as displayed in bottom right maps in Figures 1 and 3. The value of the thinnest point and its location relative to the center is displayed in the central or side box of the map.

With traditional ultrasound pachymeters, the probe needs to come into perpendicular contact with the corneal surface, and the thickness of only one point can be measured with each contact. In addition, the risk of infection and the accuracy. being user dependent are all considered significant limitations of ultrasound pachymetry. Despite these limitations, ultrasound pachymetry is the gold standard for measuring corneal thickness and is used as the basis of comparison to all other forms of pachymetry.

**Figure 5:**
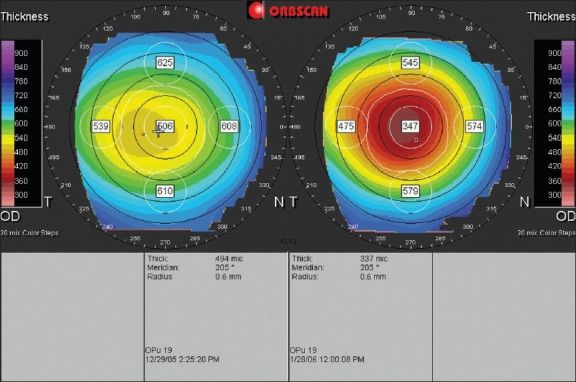
A dual pachymetric display of a right eye before (left) and after (right) keratorefractive surgery for myopia. The preoperative central corneal thickness with the Orbscan was 506 µm, compared to 490 µm with an ultrasound pachymeter (overestimation). After surgery, the values were respectively 347 µm and 395 µm (underestimation)

With the above in mind, a great number of researchers focused on Orbscan pachymetry readings. Earlier studies found significant differences between Orbscan and ultrasound readings, and while the manufacturer suggested using 0.92 as the acoustic factor in the equation to transform Orbscan readings into their ultrasound equivalents, researchers proposed a variety of other equations.^21^–^24^. Further studies indicated that a single equation was not sufficient to serve the purpose,^25^ and that Orbscan tends to overestimate pachymetry readings in thicker corneas and underestimate in thinner corneas.^26^ Technically, the corneal thickness is calculated from the elevation difference between the anterior and posterior corneal surfaces, and assuming the postoperative artifacts in posterior elevation maps described above, pachymetry can be even less accurate after refractive surgery [Figure 5].

### Corneal curvature and power

When examining an Orbscan quadmap, the corneal power map is probably the most familiar, especially when the display is Placido-based. On the axial map, topography patterns are grouped as round, oval, symmetric bow tie, asymmetric bow tie, and irregular,^6^,^27^ which are known to the experienced ophthalmologist. Studies have tested the accuracy of Orbscan measurements of the corneal curvature on calibrated test surfaces, normal subjects, and post refractive surgery cases. These studies suggest that Orbscan has acceptable accuracy in measuring the anterior and posterior corneal surface curvature in normal eyes, and thus the criteria used with traditional topography systems would be applicable to Orbscan.

**Figure 6:**
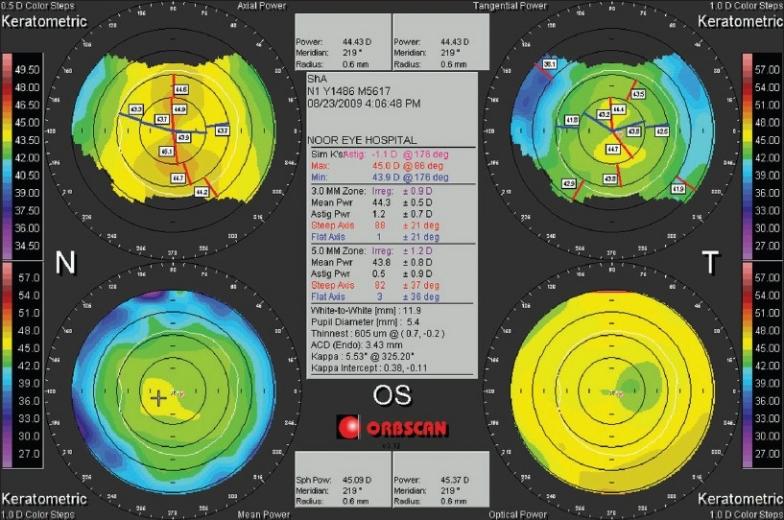
An Orbscan quadmap of an astigmatic left eye. The four maps, clockwise, from the top left include the keratometric axial, tangential, optical, and mean power. In this same order, note the decrease in the axial bias and bow pattern

In addition to traditional axial and tangential corneal power maps, the Orbscan offers displays of mean, astigmatic, and optical power maps as well. A comparison of these different options is presented in Figure 6. Many studies have focused on determining the corneal power after corneal refractive procedures using different methods and various data from the Orbscan, and some recommend using the central 4.0 mm zone of the total optical power map for this purpose.^28^,^29^ However other researchers believe that Orbscan measurements of the corneal curvature, especially the posterior corneal surface, can be erroneous in eyes that have undergone refractive surgery.^19^,^20^

**Figure 7:**
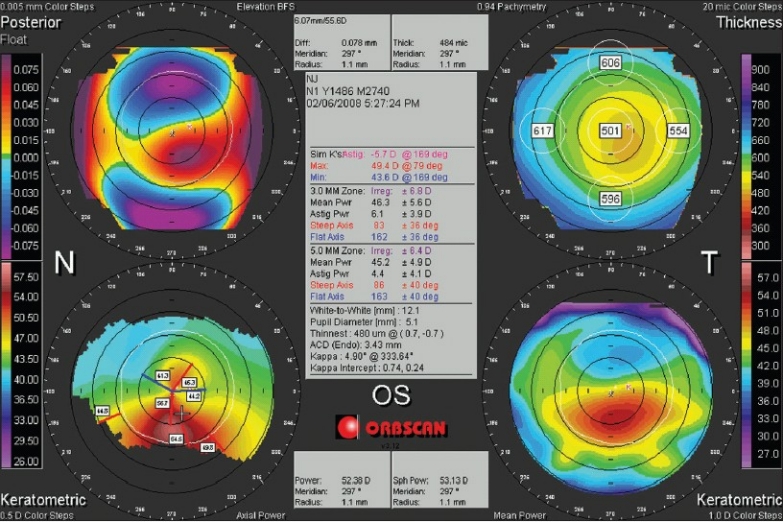
An Orbscan quadmap of the keratoconic eye in Figures 2 and 3. Note that the location of the highest point on the posterior corneal surface (top left) corresponds with the thinnest point on the pachymetry map (top right), and the steepest point of the cornea in the mean power map (bottom right). The keratometric axial power map (bottom left) shows an asymmetric bow tie, and fails to locate the cone accurately. Also note that the axial power map here is different from that in Figure 3, because it is based on Placido data

For the mean power map, the average curvature is calculated at each point on the corneal surface, and is free of the bias seen with axial and tangential curvature measurements. Since each point is defined independently, the map can demonstrate the true location of surface anomalies such as the cone in keratoconus [Figure 7].

### Corneal irregularity

The center box on the Orbscan quadmap displays the corneal irregularity index [Figures 1, 3, 6 and 7]. The Orbscan uses an algorithm to calculate the corneal irregularity indices in the 3.0 and 5.0 mm zones, which is proportional to the standard deviation of surface curvature. The index can be important in screening patients because irregularity can be associated with loss of best corrected vision that cannot be corrected with sphero-cylindrical correction. The indices were found to be strongly correlated with the maximum keratometry readings.^30^ Higher values of the index are indicative of irregular astigmatism and/or higher order aberration, and a threshold of 1.5 D for the 3.0 mm zone and 2.0-3.0 D for the 5.0 mm zone can be suggestive of keratoconus, but the index should only be used in conjunction with other findings.^31^,^32^ The irregularity indices of the 3.0 mm and 5.0 mm zones of the keratoconic eye in Figures 2 and 3 are 6.8 D and 6.4 D, respectively.

Overall, the Orbscan has user-friendly software, and its capabilities are by no means limited to what we discussed here. Nonetheless, users can be confident about results of preoperative screening tests, and acquisitions from corneas with ocular conditions, although postoperative data may be less reliable. For the Pentacam, which is another elevation-based non-contact topographer/pachymeter, we will try to maintain a similar order for the review to facilitate comparison of these two systems.

## PENTACAM

The Pentacam was originally introduced as an anterior segment analyzer that utilizes the Scheimpflug photography technique. The measurement principle dates back to 1906 when Theodor Scheimpflug was involved in aerial photography and devised this technique to enhance the quality of photographs taken from an angle. In the newer version, namely the Pentacam HR, the resolution of the photographs has improved, and data from 138 thousand points are processed.

During acquisition with the Pentacam, which can take up to 2 seconds, a rotating Scheimpflug camera photographs cross-sections of the anterior segment which are illuminated by slit lights at different meridians. Since all these slits overlap in the center of the cornea, the accuracy of central measurements is increased. The software processes data from all points and reconstructs a three dimensional representation of the anterior segment and generates readings of different parameters.

**Figure 8:**
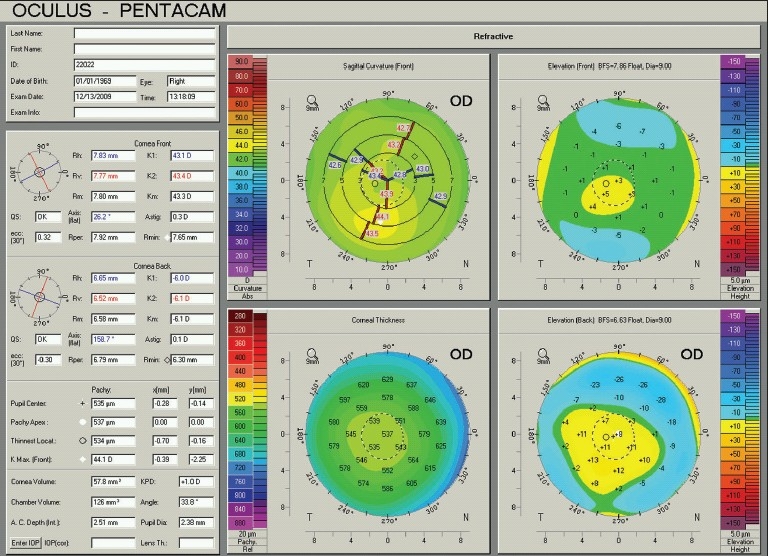
A Pentacam refractive 4-map of the same normal right eye in Figure 1. The four maps, clockwise, from the top left include the sagittal power, anterior (front) elevation, posterior (back) elevation, and pachymetry. See text for a more detailed description

The Pentacam shares many of the capabilities of the Orbscan and measures basic corneal features such as elevation, thickness and curvature. The Pentacam also displays them in the same color-coded fashion; green, yellow, and light blue for near normal values, and red and purple for caution, and the most common display is a 4-map display [Figure 8]. Figure 8 presents the Refractive 4-map display, where clockwise from the top left, the sagittal power, anterior (front) elevation, posterior (back) elevation, and pachymetry maps are included. The top left data box contains patient and exam data. Boxes underneath display quantitative data regarding the anterior and posterior corneal surfaces: Simulated keratometry readings (k1, k2) and radii of curvature (Rh, Rv), mean keratometry (Rm) and radius of curvature in the 3.0 mm zone (Km), the quality specification of the examination (QS), the axis of the flat meridian and amount of astigmatism (Astig), the mean eccentricity value in 30 degrees (ecc), the mean radius of curvature of the 7.0-9.0 mm ring area (Rper), and the minimum radius of curvature (Rmin). Pachymetry data of the center, apex, thinnest point, and their locations are followed by maximum curvature amount and location. Bottom boxes display the values of the corneal volume, keratometric power difference (KPD), chamber volume, the smaller angle size in the horizontal meridian, anterior chamber depth, and pupil diameter. The intraocular pressure (IOP) box is provided to compute the corrected IOP. Lens thickness (final box) contains a figure only when the pupil is sufficiently dilated.

Some additional features are densitometry in the Scheimpflug image display, Zernike analysis, phakic intraocular lens (PIOL) simulation and the Holladay report. The system incorporates artificial intelligence; it generates displays and calculates a number of indices that facilitate the patient screening process, which we discuss in following sections.

### Surface elevation maps

**Figure 9:**
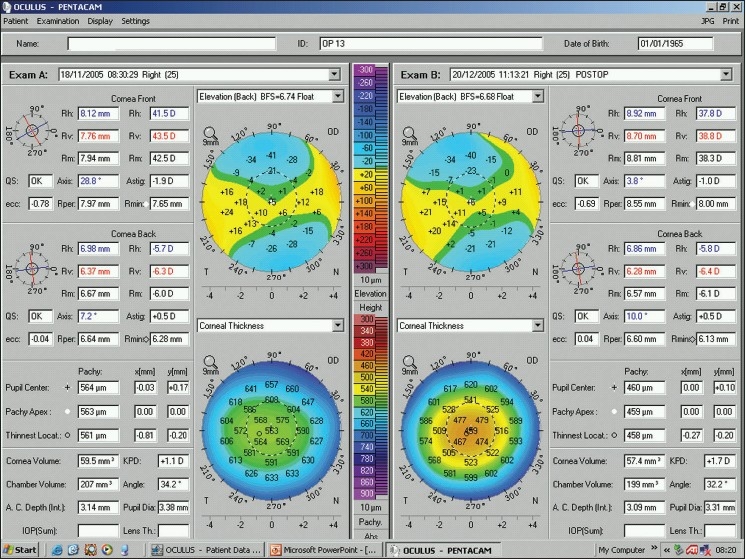
A Pentacam dual display of the same right eye in Figure 4 on the same days before (left) and after (right) keratorefractive surgery for myopia. Note the similarity in posterior corneal elevation maps (top), and the change in corneal thickness maps (bottom) as a result of central ablation

Similar to the Orbscan, anterior and posterior surface elevation measurements are plotted against a reference surface (Figure 8, top right and bottom right maps, respectively). However, normal ranges and cut-points differ between these two devices. For the anterior surface elevation, central readings less than 10-12 µm are considered normal, values greater than 15 µm could be indicative of keratoconus, and those in between fall in the grey zone. Cut-points for the posterior surface elevation are about 2-5 µm higher than the anterior surface elevation values.^33^,^34^. Overall, acceptable readings with the Pentacam are lower than that with the Orbscan. Also, in contrast to findings with the Orbscan, studies with the Pentacam indicate that keratorefractive surgery, whether surface ablation or LASIK, has no significant effect on the posterior cornea [Figure 9].^19^,^35^–^37^

**Figure 10:**
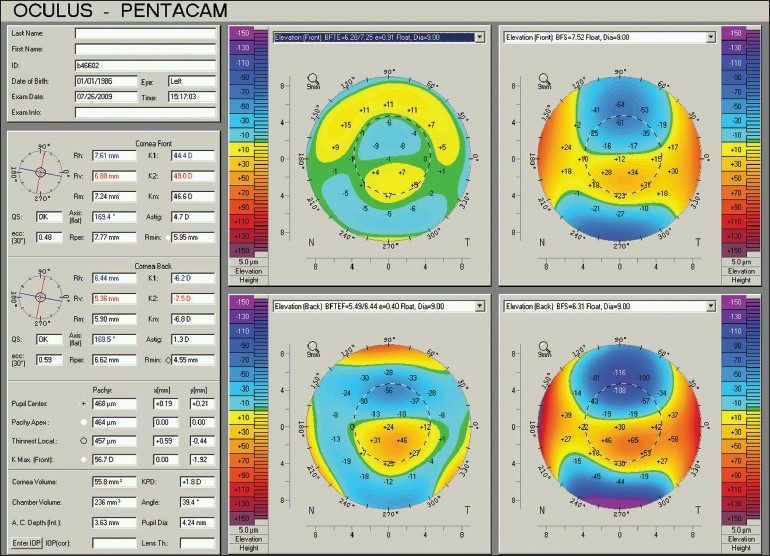
A Pentacam 4 map of the same left keratoconic eye. Note that applying a best fit toric ellipsoid reference surface to the anterior (top left) and posterior (bottom left) elevation map eliminates the toric effect of corneal astigmatism and gives a better demonstration of the exact location of abnormally heightened areas

To identify local areas of abnormality, experts believe that the shape of the normal cornea is closer to a toric ellipsoid shape than a sphere, and thus recommend using a “best fit toric ellipsoid” as the reference surface for measuring height data. This helps eliminate normal elevations seen above or below the common BFS, especially in cases of high astigmatism, and facilitates detecting irregularities. In this setting, posterior corneal elevations that exceed 15 µm above the toric ellipsoid reference surface can be considered significant [Figure 10].^38^,^39^

In another approach, elevation values on the anterior and posterior corneal surfaces are computed relative to an “enhanced reference surface”. First data from the 3.5 mm optical zone, centered on the thinnest point of the cornea, is excluded, and then the BFS for the central 8.0 mm zone is determined. The software then compares elevation readings with the standard BFS against those with the enhanced BFS. Differences between these values can indicate an abnormality, and help differentiate normal from ectatic conditions.^40^,^41^ These calculations are reflected as color-coded maps in the Belin/Ambrósio Enhanced Ectasia Display which is designed to provide a more sensitive and comprehensive evaluation.

### Corneal thickness

Using the data from the two corneal surfaces, the Pentacam determines the thickness of the cornea at all points. The system has proven to generate highly repeatable and reproducible pachymetry readings compared to ultrasound pachymeters.^42^,^43^ Pentacam measurements of the corneal thickness are comparable with ultrasound readings, and the agreement between Pentacam and ultrasonic readings is better than that for the Orbscan, especially in post-surgical eyes.^42^–^47^

**Figure 11:**
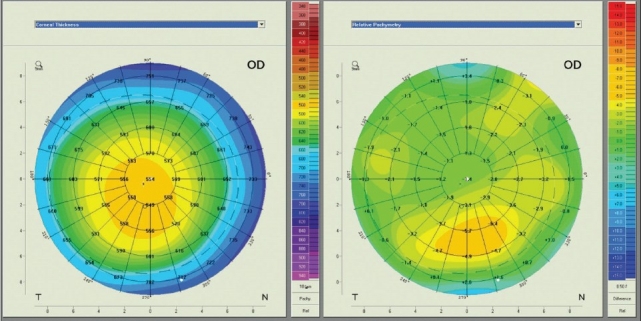
Two Pentacam single pachymetry maps of a single right eye. The actual corneal thickness map (left) demonstrates that the thinnest point is 549 µm thick, and only 5 µm thinner than the center of the cornea. The relative pachymetry map (right) shows that the abnormal area is located slightly more inferiorly, and is about 6.0% thinner than normal

In terms of display, the Pentacam generates color-coded pachymetry maps that we usually incorporate in the 4-map displays. Similar to the Orbscan, numeric values can be overlaid to facilitate reviewing the maps. On the side box, the thickness values and locations of the pupil center, apex, and thinnest point of the cornea are presented. A novel display option, however, is the relative pachymetry map, in which corneal thickness data is generated using a toric ellipsoid reference surface. On this map, the thickness of any given point is relative to the normal thickness at that point in percentages. This means that a normal map shows 0% at all points, even though the thickness increases in the periphery. When studying the relative pachymetry map, values beyond —3.0% require attention^38^ [Figure 11].

**Figure 12:**
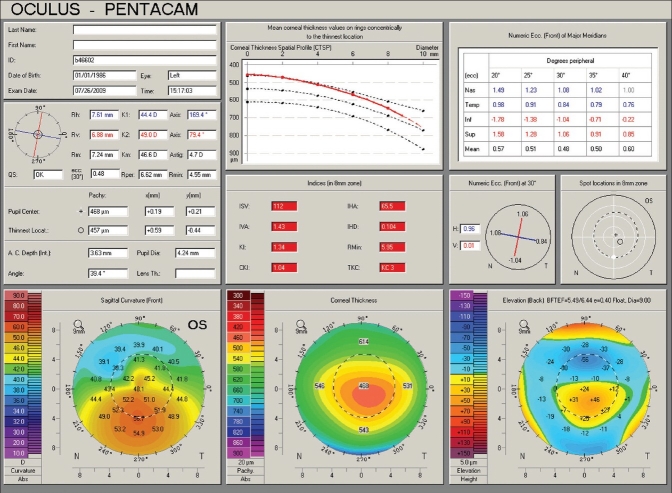
A Pentacam Refractive display of the same keratoconic left eye in Figures 3 and 10. The top middle graph demonstrates the corneal thickness spatial profile. The pachymetry at the thinnest point is 457 µm and the increase in more peripheral areas (red curve) fails to move parallel to normal (black dotted curves). Compared to 4-map displays, additional information include the value of different indices provided in the central box, and the eccentricity values along major meridians (top right). According to values of different indices, keratoconus in this eye is classified as stage 3 (KC3)

**Figure 13:**
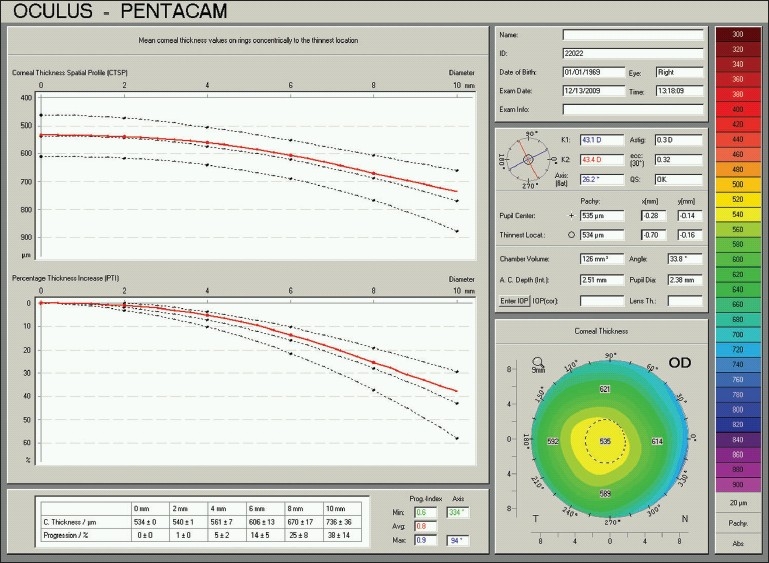
A Pentacam Pachymetric display of the same normal right eye in Figures 1 and 8. The corneal thickness at the thinnest point is 534 µm and it increases in the periphery following the normal pattern. The bottom table gives corneal thickness values at different rings and its progression in percentages

The corneal thickness spatial profile and the percentage increase in thickness from the thinnest point of the cornea towards the periphery are different between keratoconic and normal corneas.^48^ The Pentacam software uses such analysis in the “Refractive” and “Pachymetric” displays to help identify ectatic disorders and differentiate them from changes seen after keratorefractive surgery [Figures 12 and 13].

### Corneal curvature and power

As mentioned before, the Orbscan II is equipped with a Placido disc and can measure the corneal curvature directly. The Pentacam, however, is only elevation-based and thus curvature and power data are derived from elevation data. Corneal power display options with the Pentacam include the sagittal (axial) and tangential curvature of the anterior and posterior corneal surfaces, as well as the true net power, the keratometric power deviation, the refractive power, and the equivalent k-reading power. In most pre-defined displays such as the topometric, refractive, and different 4-map displays, the sagittal power map is presented. However, the tangential power is known to have less axial bias and a better tool for identifying abnormally steep areas of the cornea such as a keratoconus cone. On the tangential map, a local steepness of more than 48.0 D can be indicative of keratoconus.^38^

A number of studies have tried to determine the best Pentacam power values for intraocular lens power calculation in post refractive surgery eyes. Some reports suggest using the Holladay equivalent keratometry reading (EKR) or the true net corneal power.^49^–^51^ The Holladay EKR can be accessed in the “Holladay report” display and the “Holladay EKR detail report” display options; the former displays the EKR in the 4.5 mm zone, and the latter gives values for 1.0 mm to 7.0 mm zones as well. An important part of the detailed display is the EKR distribution graph, which can be a predictor of postoperative outcome; smaller ranges and sharp peaks favor better outcomes.^38^ Nonetheless, caution is advised in the use of Pentacam data, 52,53 because despite higher accuracy, the data should only be used in particular calculation formulas such as the Holladay 2 and the BESSt formula.^54^,^55^

### Keratoconus indices

**Figure 14:**
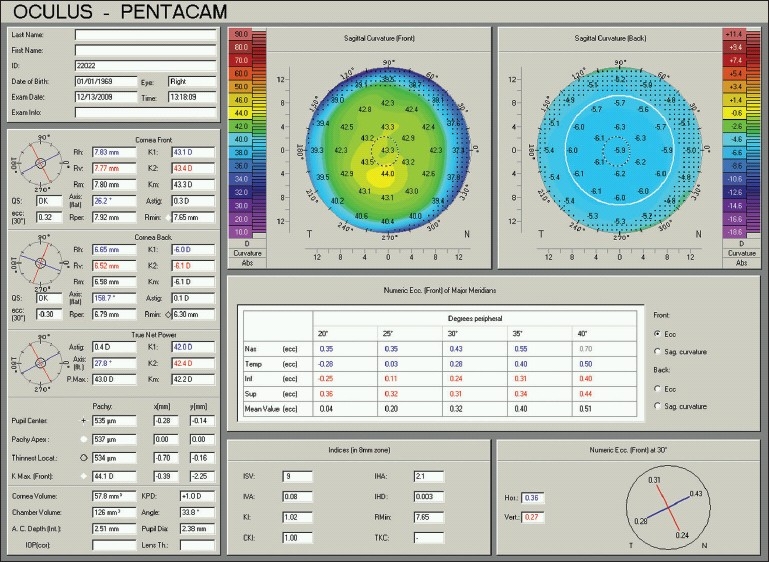
A Pentacam topometric display of the same normal right eye in figures 1, 8, and 13. The top two maps show the sagittal curvature of the anterior (left) and posterior (right) corneal surfaces. The table in the middle gives eccentricity figures in different peripheral areas and degrees. Indices are shown in the middle box in the bottom. Note that none of the indices are highlighted, as they are all within the normal range

The Pentacam uses the acquired data to compute a number of indices most of which can be found in the “Refractive” and “Topometric” displays [Figures 12 and 14]. The software highlights abnormal values and uses them to classify the stage of keratoconus. For example, the index of surface variance (ISV) is indicative of early signs when less than 30. For keratoconus stage 1 to 3, the software takes ISV ranges of 30-55, 55-90, and 90-150, respectively. ISV values over 150 are associated with a stage 4 keratoconus. For the keratoconus index (KI), the respective ranges for these 5 stages are 1.04-1.07, 1.07-1.15, 1.10-1.25, 1.15-1.45, and >1.50. Other quantitative indices computed by the Pentacam include the index of surface asymmetry (IVA), the center keratoconus index (CKI), the index of height asymmetry (IHA), the index of height decentration (IHD), the aberration coefficient (ABR), and the eccentricity.

The parameters discussed here are only some of the data generated by the Orbscan and the Pentacam. As anterior segment analyzers, they are both capable of displaying anterior chamber depth (ACD) maps, which can be useful when PIOL implantation is considered.

Reviewing different parameters is only part of the preoperative screening process, and there are a number of other issues that must be considered. Firstly, the Orbscan and the Pentacam are diagnostic tools to assist in clinical decision making. They provide an enormous amount of valuable data concerning the anterior segment and the anterior and posterior corneal surfaces in a non-contact fashion, and although they have many advantages, the user should be aware of their limitations. One such example is the postoperative acquisition with the Orbscan.

Secondly, the suggested criteria for different devices are not interchangeable, and thus the origin of data should be considered. Also, the criteria are mostly introduced to avoid potential complications of refractive surgery and in some cases they may lead to unnecessary exclusion of patients, especially if patient history and other clinical findings such as refraction, visual acuity, and slit lamp examination results are ignored. Any given patient may suffer from ocular or systemic diseases that are not detectable by the Orbscan or Pentacam maps but may be contraindicated for refractive surgery.

Although significant progress has been made over time, our dependence on traditional gold-standard reflection-based topographers is still not over. There is no doubt that the future holds even greater advances, and new technology will be continuously introduced. Continued efforts and research should improve the accuracy of these diagnostic tools, as well as the artificial intelligence constructs incorporated within these units to make the screening process more specific, more sensitive, and more reliable.
